# The Effect of Watermelon Juice Supplementation on Heart Rate Variability and Metabolic Response during an Oral Glucose Challenge: A Randomized, Double-Blind, Placebo-Controlled Crossover Trial

**DOI:** 10.3390/nu15040810

**Published:** 2023-02-04

**Authors:** Rachel Matthews, Kate S. Early, Cullen M. Vincellette, Jack Losso, Guillaume Spielmann, Brian A. Irving, Timothy D. Allerton

**Affiliations:** 1 Department of Kinesiology, Louisiana State University, Baton Rouge, LA 70803, USA; 2 Department of Kinesiology and Health Sciences, Columbus State University, Columbus, GA 39207, USA; 3 School of Nutrition and Food Sciences, Louisiana State University, Baton Rouge, LA 70803, USA; 4 Pennington Biomedical Research Center, Vascular Metabolism Laboratory, Baton Rouge, LA 70808, USA

**Keywords:** heart rate variability, watermelon, nitric oxide, L-citrulline, L-arginine, metabolism, hyperglycemia, autonomic function, indirect calorimetry

## Abstract

Heart rate variability (HRV) provides a simple method to evaluate autonomic function in health and disease. A reduction in HRV may indicate autonomic dysfunction and is strongly associated with aspects of cardiometabolic disease, including hyperglycemia. Reduced nitric oxide (NO) bioavailability is also implicated in the development of cardiometabolic disease and autonomic dysfunction. Watermelons are natural sources of L-arginine and L-citrulline, substrates used for NO synthesis. Watermelon consumption can improve NO bioavailability. We conducted a randomized, double-blind, placebo-controlled crossover trial to test the effects of 2 weeks of daily watermelon juice (WMJ) supplementation on HRV in response to an oral glucose challenge (OGC) in healthy young adults. We also performed indirect calorimetry to assess if our intervention altered the metabolic response to the OGC. WMJ supplementation preserved high-frequency power (HF) (treatment effect, *p* = 0.03) and the percentage of successive differences that differ by more than 50 ms (pNN50) (treatment effect, *p* = 0.009) when compared to the placebo treatment. There was no difference in resting energy expenditure or substate oxidation according to treatment. We report that WMJ supplementation attenuates OGC-induced reductions in HRV. Future work should emphasize the importance of NO bioavailability in autonomic dysfunction in cardiometabolic disease.

## 1. Introduction

Autonomic dysfunction is an emerging mechanism in the development of cardiometabolic disease [1,2]. A simple and reliable method of assessing autonomic dysfunction is through heart rate variability (HRV), or the variation from two consecutive heartbeats. Decreased parasympathetic activity suggests low variation in consecutive beats (i.e., low HRV), which has been linked to increased cardiovascular disease risk and mortality [1]. In addition, visceral adipose tissue accumulation [3], hyperglycemia [1,4], endothelial dysfunction [5], and increased inflammation [6] have all been associated with reduced HRV. These observational studies indicate that HRV and autonomic dysfunction are likely included in the pathology of obesity and type 2 diabetes mellitus (T2DM). 

Deficits in energy expenditure and substrate utilization are also aspects of autonomic dysfunction that characterize cardiometabolic diseases. During meal feeding or insulin stimulation, hormonal and sympathovagal coordination regulates blood flow and metabolic flux. In healthy men, postprandial energy expenditure correlates with sympathetic nervous system activity [6,7]. In patients with T2DM, fasting, and postprandial blood glucose negatively correlate with HRV [4,8]. Likewise, patients with obesity have an attenuated HRV response to insulin infusion compared to lean, age-matched controls [9]. When combined with the known metabolic dysfunction accompanying obesity and T2DM, a reduction in HRV may serve as an early signal of impending autonomic-metabolic decline. 

An oral glucose challenge (OGC) has been shown to reduce HRV in diverse patient populations [10,11]. The transient hyperglycemia experienced during an OGC can also reduce vascular endothelial function, ischemic reperfusion, and microvascular blood flow [12,13,14]. Hyperglycemia-induced oxidative stress is thought to reduce nitric oxide (NO) bioavailability, leading to these impairments. The amino acid L-arginine serves as the substrate for NO synthase to promote the enzymatic formation of NO. In healthy participants, increasing the plasma concentrations of L-arginine resulted in improved vagal control of heart rate [15]. Other NO-promoting therapies, such as nitrates and beetroot juice, have also shown some promise in improving HRV [16,17]. The amino acid L-citrulline, when consumed orally, is rapidly converted to L-arginine by a partial urea cycle in the kidney [18]. Compared to oral consumption of L-arginine, L-citrulline increases plasma concentrations to a greater degree [19]. Oral L-arginine is subject to a high degree of hepatic extraction and conversion to urea and L-ornithine. Whereas, L-citrulline (minimal effective dose ~3 g) is not subject to the same degree of first-pass extraction and promotes NO formation by direct activation of NO synthase enzyme and by its conversion to L-arginine [18]. Watermelons are an abundant natural source of L-citrulline and L-arginine [18,20]. Furthermore, regular consumption of watermelon juice (WMJ) for 2 weeks can increase NO bioavailability [21]. We have previously reported that WMJ supplementation attenuated some aspects of vascular decline during an OGC [22]. 

The loss of NO bioavailability and reduction in HRV during an OGC suggests that these phenomena may be causally linked. To date, no studies have explored the protective effects of NO donor therapy on hyperglycemia-induced autonomic dysfunction and metabolic function. In a randomized, double-blind, placebo-controlled crossover trial, we studied the effects of two weeks of WMJ supplementation on HRV and metabolic control during an oral glucose challenge (OGC). We hypothesized that increased NO bioavailability via WMJ supplementation would attenuate the decline in HRV during brief, transient hyperglycemia. We also sought to evaluate our intervention’s impact on metabolic parameters (energy expenditure and substrate oxidation) using indirect calorimetry. 

## 2. Materials and Methods

### 2.1. Participants and Study Design

**Figure 1:**
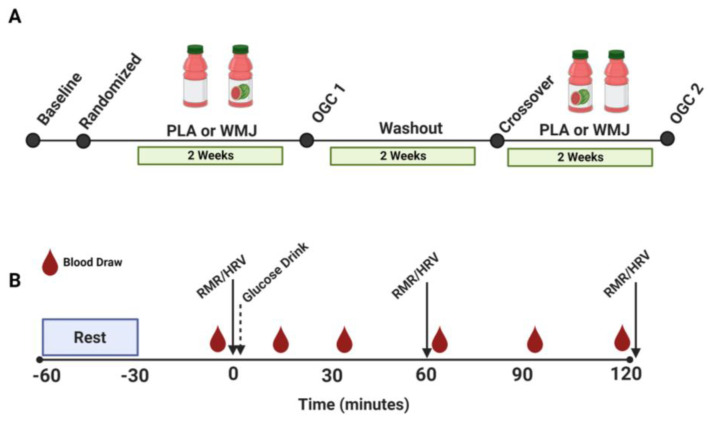
(**A**) Participant randomization and train schedule for two treatments (WMJ and PLA). (**B**) Study visit sampling time course for RMR, HRV, and blood draws during the OGC study visit conducted after the 2-week WMJ or PLA treatment period. WMJ, watermelon juice; PLA, placebo; RMR, resting metabolic rate; HRV, heart rate variability; OGC, oral glucose challenge.

Before enrollment, eligible participants provided written and informed consent. Our participation criteria were based on age (18–40 years of age) with a BMI between 18 and 29.9 kg/m^2^. We excluded participation based on the presence of type 1 or type 2 diabetes mellitus, cardiovascular disease, or any allergies to watermelon. Study participants completed a pre-randomization visit to obtain baseline characteristics. Subsequently, participants were randomized to complete 2 weeks of daily WMJ supplementation (500 mL) or a placebo drink (PLA). After the 2-week supplementation period, participants returned to the study site to complete a 75-g OGC (Figure 1A). During the OGC (Figure 1B), HRV, resting energy expenditure, and respiratory exchange ratio (RER) (via indirect calorimetry) were measured for 30 min before the glucose drink (0 min) and 60 min and 120 min after the glucose ingestion. After completing the first intervention, participants crossed over to the opposite intervention and completed a second OGC. A 2-week washout period separated the first and second treatments. The details on the WMJ supplement regiments and the nutritional content of the WMJ and PLA drinks, and the primary study findings have been reported elsewhere [22]. The study was approved by the Pennington Biomedical Research Center and Louisiana State University Institutional Review Board. This trial was registered at clinicaltrials.gov under NCT04092439.

### 2.2. Oral Glucose Challenge (OGC)

Participants arrived in the morning at approximately 6:30 AM in the fasted state and abstained from alcohol, exercise, and caffeine at least 48 h before the study visit. A baseline (0-min) blood sample was collected from a flexible intravenous catheter inserted into the antecubital vein of the left arm. After participants drank a 75 g glucose beverage (Glucola, Mercedes Scientific), blood samples were collected at 15, 30, 60, 90, and 120 min. Blood was collected for the catheter at each time point in an EDTA tube and centrifuged. Plasma glucose was then measured (Analox GL5, Analox Instruments Ltd., Stourbridge, United Kingdom).

### 2.3. Heart Rate Variability (HRV)

A Zephyr BioHarness strap and BioModule sensor (Medtronic, Boulder, CO) were used to continuously monitor electrographic (ECG) and respiratory data at a sampling frequency of 1000 Hz. All participants were supine with free breathing for a stationary assessment of heart rate for 5 min. From this, the RR intervals were quantified, and HRV was determined (Kubios HRV, Version 3.4.1, Kuopio, Finland).

HRV was analyzed in time and frequency domain measures [23,24]. Time domain parameters included mean RR intervals (RR), the standard deviation of RR interval (SDNN), root mean square of standard deviation (RMSSD), and the percent of RR intervals with a difference in successive RR intervals longer than 50 ms (pNN50). SDNN is a reflection of short-term overall variability (both sympathetic and parasympathetic activity), whereas RMSSD is suggestive of parasympathetic activity. Frequency domain parameters were calculated using the fast Fourier transform (FFT) and included low frequency (LF) (0.04–0.15 Hz), high frequency (HF) (0.15–0.4 Hz), total power (TP), and the low-frequency-to-high-frequency ratio (LF/HF). HF relates to parasympathetic activity and is correlated to pNN50 and RMSSD time domain measures [25]. The LF band mainly reflects baroreceptor activity, with sympathetic and parasympathetic activity contributing [24]. Lastly, a low LF/HF ratio reflects parasympathetic dominance [24,26].

### 2.4. Indirect Calorimetry

In conjunction with the oral glucose challenge, indirect calorimetry was performed using a ventilated hood (ParvoMedics, Inc., Sandy, UT) to determine the expired volume of oxygen (VO2) and carbon dioxide (VCO2). Briefly, participants arrived in a fasted state and were instructed to rest supine for 30 min with the lights dimmed to ensure a rested state. Then a ventilated hood was placed over the participant’s head and shoulders. Exhaled air was continuously measured for the 30 min preceding glucose ingestion (0 min) to measure resting energy expenditure (REE) and substrate oxidation. We repeated these measurements at the 60 min and 120 min time points. REE, RER, carbohydrate, and fat oxidation were calculated according to previously published equations [27]. 

### 2.5. Statistical Analysis

Distribution and normality were assessed by a Shapiro–Wilk test. A linear mixed model was used to assess differences between treatment, time points (0, 60-, and 120-min), and visit order (fixed effects). Subject ID was treated as a random effect. Time points, treatment, and interactions were adjusted for visit order and tested using a linear mixed effects model. Post hoc differences were determined by a Student’s t-test. A linear regression was performed to calculate Pearson’s correlation coefficient. Data are reported as means ± SD. Statistical significance was determined as a *p*-value < 0.05.

## 3. Results

### 3.1. Participant Characteristics

Table 1 provides baseline participant characteristics. We studied 18 healthy young men and women. Participants had a normal BMI (<25.0 kg/m^2^), were non-smokers, and were recreationally active. 

### 3.2. HRV

There were no significant treatment effects of glucose across time points (*p* = 0.08) or glucose area under the curve (*p* = 0.43). Table 2 provides the data and statistical analysis for HRV parameters. Heart rate (HR) increased throughout the oral glucose challenge (Time effect < 0.0005) but to an equivalent degree for WMJ and PLA treatment (treatment effect *p* = 0.68). SDNN and RMSSD were significantly affected by the oral glucose challenge (time effect, *p* = 0.007 and *p* = 0.024, respectively) but not by treatment (*p* = 0.72 and *p* = 0.17, respectively). The percentage of successive differences that differ by more the 50 ms (pNN50) was increased during WMJ treatment (treatment effect *p* = 0.009). Time domain components for low frequency (LF) and high frequency (HF) were both reduced significantly during the OGC (time effects, *p* = 0.02 and *p* = 0.007, respectively). However, WMJ significantly (treatment effect, *p* = 0.03) attenuated the decline in HF (ms2) as plasma glucose increased. The glucose challenge reduced total power (time effect, *p* = 0.013). A near-significant interaction (*p* = 0.051) was detected for the ratio of LF to HF (LF/HF).

### 3.3. Metabolic Parameters

**Figure 2:**
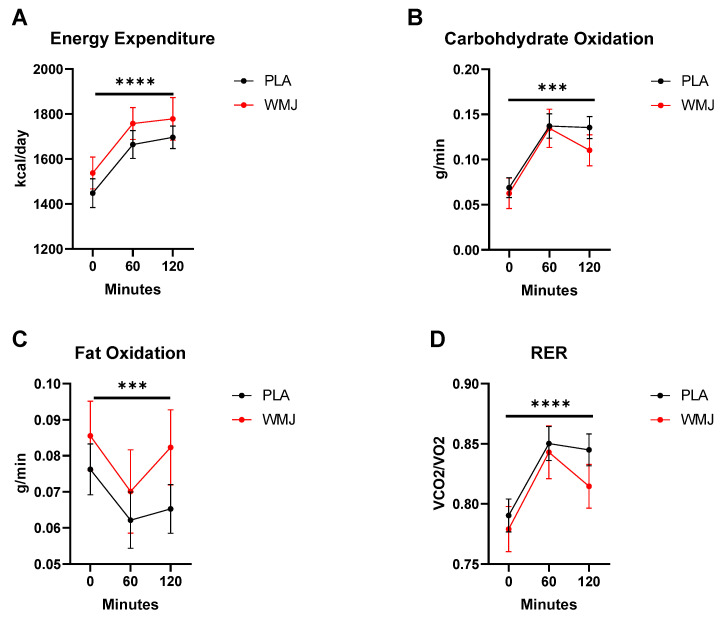
Metabolic responses comparison between PLA versus WMJ treatment during the OGC. (**A**) Energy expenditure, (**B**) carbohydrate oxidation (CHO Ox), (**C**) fat oxidation (Fat Ox), and (**D**) respiratory exchange ratio (RER) all increased significantly during the OGC. No significant treatment effects were detected. Data are means ± SD. (time effects indicated as *** *p* < 0.001 and **** *p* < 0.0001.)

As anticipated, several metabolic parameters were elevated in response to the glucose challenge. Figure 2 shows that REE (*p* < 0.0001) and RER (*p* < 0.0001) were both increased during the time course of the OGC. Likewise, carbohydrate oxidation (CHO Ox) increased (time effect, *p* < 0.001), and fat oxidation (Fat Ox) decreased (time effect, *p* = 0.002) during the glucose challenge. However, no treatment effects or interactions were detected for any metabolic parameter measured. Metabolic flexibility, calculated as the difference between RER 60 min post glucose versus RER at 0 min (PLA, 0.013 ± 0.2 vs. WMJ, 0.010 ± 0.2), was not different between treatments (*p* = 0.64).

Table 3 shows Pearson’s correlation coefficients for REE and RER responses versus HRV parameters during the OGC. The REE was negatively correlated with pNN50 (r = −0.24, *p* = 0.04) and HF (r = −0.24, *p* = 0.04) and positively correlated with LF/HF ratio (r = 0.27, *p* = 0.02). There were no significant treatment effects or interactions for REE and HRV parameters. RER was negatively correlated with SDNN (r = −0.24, *p* = 0.04) and TP (r = −0.25, *p* = 0.04). There was a significant negative relationship between CHO Ox and RMSSD (r = −0.27, *p* = 0.02), SDNN (r = −0.29, *p* = 0.01), LF (r = −0.29, *p* = 0.01), HF (r = −0.25. *p* = 0.04), TP (r = −0.29, *p* = 0.01), and PNS index (r = −0.29, *p* = 0.01) while there was a positive correlation with the SNS index (r = 0.36, *p* < 0.01) and stress index (r = 0.30, *p* = 0.01). There were no significant correlations between Fat Ox and the HRV parameters. Linear regression analysis did not detect any significant treatment effects for HRV and REE, RER, CHO Ox, or Fat Ox.

## 4. Discussion

Hyperglycemia attenuates HRV by reducing parasympathetic tone and/or increasing sympathetic nervous system activity [1,4,28]. Sustained and acute hyperglycemia also reduces NO bioavailability, which directly and indirectly impacts HRV by altering vagus nerve activity [29,30,31,32]. Watermelon is a natural source of L-arginine and L-citrulline that can improve NO bioavailability [20,21]. Our previous work demonstrated that WMJ supplementation attenuated aspects of macro- and microvascular dysfunction induced by the OGC [22]. In the current study, we demonstrate that WMJ supplementation preserves aspects of autonomic function during acute hyperglycemia (OGC) but does not alter metabolic responses when compared to PLA treatment. 

We confirm that in healthy young participants, an OGC reduces HRV, as evidenced by the reduction in SDNN, RMSSD, LF, HF, and TP (Table 2). Hyperglycemia promotes oxidative stress and reduces NO bioavailability [33,34,35]. We and others have reported a reduction in vascular endothelial function during an OGC [22,33]. In addition, in agreement with other reports, we show that total variability and parasympathetic activity (RMSSD and HF) were significantly reduced during the OGC. However, supplemental WMJ preserved parasympathetic activity as indicated by treatment effects for pNN50, RMSSD, and HF (Table 2). In a previous study by Massa et al. (2016), 6 weeks of watermelon extract supplementation was not effective at improving HRV in prehypertensive and hypertensive adults [36]. Similarly, we also did not observe improvements in fasted, baseline HRV between PLA and WMJ treatment. However, the OGC is clearly a perturbation that induces autonomic dysfunction. The aforementioned study only treated participants with prehypertension and hypertension but not metabolic dysfunction. In a group of participants with elevated risk for cardiometabolic disease, obese postmenopausal women, 8 weeks of L-citrulline supplementation improved HRV [37]. Therefore, hyperglycemia and oxidative stress may be necessary mediators for reduced HRV.

The OGC is also a metabolic perturbation that causes an increase in REE and a shift in substrate oxidation. In the fasted state, fat is the primary substrate utilized. In the postprandial state, increased plasma glucose and insulin are cues for metabolism to shift from fat to carbohydrate oxidation. In response to a meal challenge or insulin stimulation, REE and RER are positively associated with sympathetic activity [7,9]. As previously stated, the OGC increases REE, RER, and CHO Ox, while reducing Fat Ox. There were no treatment effects for energy expenditure or substrate utilization during OGC (Figure 2). We report only modest and mostly negative correlations between HRV and REE, RER, and CHO Ox (Table 3). No significant correlations were found when comparing HRV to Fat Ox. Again, our participants were young, healthy, and cleared the glucose drink as expected. Therefore, under this circumstance, the OGC is sufficient to reduce vascular and autonomic function but not metabolic control of energy expenditure and substrate oxidation. This strengthens the argument that vascular and autonomic dysregulation precedes the development of whole-body metabolic impairments [11,38,39,40,41]. Evaluation of participants with obesity and existing metabolic dysfunction may provide more insight into the connection between autonomic and metabolic regulation. 

A major limitation of our study is our inability to directly link improved NO bioavailability to the preservation of HRV during the OGC. Several studies have documented the importance of NO to autonomic function in health and disease [15,29,31,32]. Our group has shown that the OGC reduces NO-mediated vasodilation that can be attenuated with WMJ supplementation [22]. However, watermelon is also a rich source of antioxidants vitamin C and lycopene, which can reduce oxidative stress and indirectly improve NO bioavailability [42,43]. Other limitation includes a relatively low sample size and the inability to determine the impact of sex on our results. Despite these limitations, we add to the literature that the OGC is a sufficient metabolic perturbation to reduce autonomic dysfunction in healthy individuals without impacting systemic metabolic parameters. 

In conclusion, we show that the autonomic system is susceptible to a hyperglycemic episode. Furthermore, using a rigorous study design, we show the efficacy of a naturally rich source of amino acids, L-citrulline, and L-arginine, to preserve HRV during a hyperglycemic episode. These findings build on our previous work that shows WMJ supplementation protects vascular function during hyperglycemia. NO bioavailability is potentially a link between these two integrated physiological systems, but more work is required to develop a mechanistic understanding of this relationship. 

## Figures and Tables

**Table 1:** Baseline participant characteristics.

Sex	Males (*n* = 6), Females (*n* = 12)
Age (years)	23.6 ± 3.1
Body weight (kg)	66.8 ± 12.3
BMI (kg/m^2^)	23.6 ± 3.1

Values are means ± SD.

**Table 2:** Heart rate variability response during the oral glucose challenge.

Time Points	0		60		120		*p*-Values		
Parameter	PLA	WMJ	PLA	WMJ	PLA	WMJ	Time	Treatment	Interaction
Plasma Glucose (mmol/L)	5.19 ± 0.38	5.24 ± 0.36	7.05 ± 2.15	7.83 ± 1.95	5.22 ± 1.42	5.88 ± 1.67	<0.0001 *	0.0869	0.4063
HR (bpm)	64.3 ± 13.2	66.9 ± 15.4	68.5 ± 13.5	69.3 ± 17.9	70.7 ± 13.6	74.0 ± 21.6	0.0005 *	0.6866	0.6570
RR (ms)	969.9 ± 195.5	933.6 ± 176.8	909.2 ± 188.2	908.1 ± 186.6	879.0 ± 177.6	858.6 ± 201.0	0.0002 *	0.9153	0.5509
SDNN (ms)	67.7 ± 47.9	76.5 ± 37.5	50.1 ± 24.0	56.7 ± 28.2	58.6 ± 32.7	61.4 ± 35.3	0.0070 *	0.7274	0.6777
RMSSD (ms)	73.9 ± 57.0	90.6 ± 54.0	52.8 ± 27.7	74.0 ± 46.8	54.0 ± 31.9	77.1 ± 56.0	0.0243 *	0.1749	0.9207
pNN50 (%)	30.8 ± 26.7	43.4 ± 28.2	27.1 ± 23.4	42.2 ± 30.3	21.4 ± 22.6	35.5 ± 35.1	0.1379	0.0099*	0.5850
LF (ms^2^)	2525 ± 2944	2234 ± 2478	985 ± 1006	1073 ± 802	1813 ± 1543	2222 ± 2052	0.0209 *	0.8782	0.6531
LF (nu)	50.4 ± 14.1	44.7 ± 24.0	46.9 ± 18.0	45.5 ± 25.4	55.2 ± 18.4	54.1 ± 25.1	0.1480	0.7886	0.3320
HF (ms^2^)	2696 ± 3204	4205 ± 3636	1356 ± 1273	2560 ± 2755	1511 ± 1630	3212 ± 3378	0.0075 *	0.0394 *	0.6888
HF (nu)	49.5 ± 14.1	55.3 ± 24.0	53.1 ± 18.0	54.4 ± 25.4	44.8 ± 18.4	45.9 ± 25.1	0.1493	0.7892	0.3265
TP (ms^2^)	5404 ± 6141	6642 ± 5735	2412 ± 2009	3710 ± 3179	3572 ± 2720	5610 ± 5184	0.0132 *	0.1190	0.9679
LF/HF	1.34 ± 1.36	1.50 ± 1.92	1.12 ± 0.81	1.50 ± 1.73	1.62 ± 1.11	2.06 ± 2.01	0.2383	0.1911	0.0516 *

SDNN, standard deviation of NN intervals; RMSSD, root mean square of successive RR interval differences; pNN50, percentage of successive differences that differ by more than 50 ms; LF, low-frequency power; HF, high-frequency power; TP, total power; LF/HF, ratio of LF-to-HF power. Values are means ± SDs. * *p*-value ≤ 0.05.

**Table 3:** HRV and metabolic parameter correlations.

	REE		RER		CHO Ox		Fat Ox	
Parameter	r	*p*-Value	r	*p*-Value	r	*p*-Value	r	*p*-Value
RMSSD (ms)	−0.228	0.059	−0.209	0.085	−0.278	0.020 *	0.115	0.347
SDNN index (ms)	−0.184	0.130	−0.241	0.046	−0.290	0.015 *	0.149	0.222
pNN50 (%)	−0.246	0.042	−0.089	0.468	−0.157	0.199	0.003	0.983
VLF (ms^2)^	−0.036	0.771	−0.125	0.306	−0.113	0.357	0.077	0.530
LF (ms^2^)	−0.099	0.420	−0.291	0.015 *	−0.291	0.015 *	0.195	0.109
HF (ms^2^)	−0.241	0.046 *	−0.176	0.148	−0.247	0.040 *	0.082	0.503
LF/HF ratio	0.269	0.025 *	−0.038	0.760	0.048	0.695	0.102	0.404
Total power (ms^2^)	−0.194	0.110	−0.247	0.041	−0.289	0.016 *	0.143	0.242
PNS Index	−0.160	0.190	−0.244	0.043 *	−0.296	0.013 *	0.167	0.170
SNS Index	0.095	0.437	0.292	0.010 *	0.326	0.006 *	−0.227	0.061
Stress Index	0.110	0.369	0.271	0.024 *	0.303	0.011 *	−0.200	0.100

SDNN, standard deviation of NN intervals; RMSSD, root mean square of successive RR interval differences; pNN50, percentage of successive differences that differ by more than 50 ms; LF, low-frequency power; HF, high-frequency power; TP, total power; LF/HF, ratio of LF-to-HF power. Values are means ± SDs. * *p*-value ≤ 0.05.

## Data Availability

The data contained in the manuscript are available upon request to the corresponding author.
